# Translation and psychometric properties of the Chinese version of the Leeds Attitudes to Concordance II scale

**DOI:** 10.1186/s12911-015-0184-0

**Published:** 2015-08-01

**Authors:** Wei He, Ann Bonner, Debra Anderson

**Affiliations:** School of Nursing, Nantong University, No. 19 Qixiu Road, Chongchuan District, Nantong City, Jiangsu Province People’s Republic of China 226001; School of Nursing, Queensland University of Technology, Victoria Park Rd, Kelvin Grove, QLD Australia 4059; Kidney Health Service, Royal Brisbane & Women’s Hospital, Herston, QLD Australia 4059

**Keywords:** Concordance, Health communication, Scale, Translation, Psychometric properties, China

## Abstract

**Background:**

Concordance is characterised as a negotiation-like health communication approach based on an equal and collaborative partnership between patients and health professionals. The Leeds Attitudes to Concordance II (LATCon II) scale was developed to measure the attitudes towards concordance. The purpose of this study was to translate the LATCon II into Chinese and psychometrically test the Chinese version of LATCon II (C-LATCon II).

**Methods:**

The study involved three phases: i) translation and cross-cultural adaptation; ii) pilot study; and iii) a cross-sectional survey (*n* = 366). Systematic random sampling was used to recruit hypertensive patients from nine communities covering around 78,000 residents in China. Tests of psychometric properties included content validity, construct validity, criteria-related validity (correlation between the C-LATCon II and the Therapeutic Adherence Scale for Hypertensive Patients (TASHP)), internal reliability, and test-retest reliability (*n* = 30).

**Results:**

The study found that the C-LATCon II had a satisfactory content validity (item-level Content Validity Index (CVI) = 0.83-1, scale-level CVI/universal agreement = 0.89, and scale-level CVI/averaging calculation = 0.98), construct validity (four components extracted explained 56.66 % of the total variance), internal reliability (Cronbach’s alpha of overall scale and four components was 0.78 and 0.66-0.84, respectively), and test-retest reliability (Pearson’s correlation coefficient = 0.82, *p* < 0.001; interclass correlation coefficient = 0.82, *p* < 0.001; linear weighted kappa statistic for each item = 0.40-0.65, *p* < 0.05). Criteria-related validity showed a weak association (Pearson’s correlation coefficient = 0.11, *p* < 0.05) between patients’ attitudes towards concordance during health communication and their health behaviours for hypertension management.

**Conclusions:**

The C-LATCon II is a validated and reliable instrument which can be used to evaluate the attitudes to concordance in Chinese populations. Four components (health professionals’ attitudes, partnership between two parties, therapeutic decision making, and patients’ involvement) describe the attitudes towards concordance during health communication.

**Electronic supplementary material:**

The online version of this article (doi:10.1186/s12911-015-0184-0) contains supplementary material, which is available to authorized users.

## Background

The concept of concordance originates from a focus on a medical problem: “Why do patients not follow the treatment, as directed?” One of the primary reasons is that during health communication, the treatment regimens that are decided by health professionals might not be feasible or acceptable to patients. This mismatch between agreement calls for a shift of focus in health promotion from merely evaluating patients’ individual health behaviours to focusing on the earlier stage – assessing health communication between patients and health professionals.

The publication of the concordance report [1] signals a new way of thinking about health communication. Concordance is characterised as a negotiation-like health communication approach based on an equal partnership between patients and health professionals, collaborative attitude in therapeutic decision-making, and sufficient consideration of the patients’ preferences [2–4]. Concordant health communication seeks to improve the trust between patients and health professionals involved and the acceptability and feasibility of the treatment regimens, which are then likely to contribute to the level of adherence to treatment regimens [5, 6].

Raynor et al., [7] developed a 12-item Leeds Attitudes to Concordance (LATCon) scale for measuring the attitudes towards concordance during health communication. It has been used in several studies by recruiting health professionals [7], patients [8], medical students [9], and pharmacists [10]. Knapp et al., [11] subsequently modified the LATCon and developed a 20-item (Leeds Attitudes to Concordance scale II (LATCon II). The LATCon II has been used in three studies to assess health professionals’ attitudes towards concordance [11–13].

So far, the LATCon has been translated into Finnish [10] and the LATCon II has been translated into Spanish [12]. Following permission of the LATCon II authors, this study sought to translate the LATCon II into Chinese and examine the psychometric properties in a Chinese population.

## Methods

### Design

This study comprised three stages: 1) structural process to translate the original English version of LATCon II into the target Chinese version of LATCon II; 2) pilot study to evaluate the participants’ understanding of the translated LATCon II; and 3) primary cross-sectional survey to test the psychometric properties of the translated LATCon II.

### Sample and settings

This study recruited hypertensive patients as the participants for two reasons. First, patients with chronic diseases are often in the best position to know what is required to manage their health on a daily basis. So, the concept of concordance aligns better within chronic health condition prevention and management settings [5, 14]. Second, hypertension is a chronic disease, which requires lifelong treatment. The inclusion criteria for recruiting participants included: 1) diagnosis of primary hypertension, 2) ≥ 18 years of age, 3) have been provided with treatment regimens for hypertension management (i.e. medication therapy and lifestyle modifications), and 4) willing to participate in the study. The exclusion criteria were: 1) secondary hypertension, 2) patients with severe complications of hypertension (e.g. stroke and severe heart failure), and 3) patients who were cognitively impaired.

Participants were recruited from nine Community Health Centres (CHCs) in Nantong City, China. Nantong City has a population of 7.6 million [15], and the nine CHCs targeted covers around 78,000 residents. While the overall prevalence of hypertension in China was estimated to be 18.8 % [16], the prevalence of hypertension in Nantong City was 21.61 % in 2013 [17]. CHCs have been established in China to provide disease prevention and control, healthcare services, health education, family planning, medical treatment services, and community rehabilitation [18]. Electronic Health Records (EHRs) are used in CHCs for recording health prevention and treatment plans for residents. In this study, EHRs assisted with identifying the potential participants who attended the CHCs.

The sample size for testing psychometric properties is recommended to be at least 10 times the number of the items in an instrument [19]. As a result, the minimum sample required for this study was 200 (20 items in the original LATCon II). We planned to recruit 300 participants in this study. Considering a possible non-response rate of 25 %, we aimed to recruit 400 (300/75 %) potential participants. At the beginning of the data collection, 3,287 hypertensive patients were available in the EHRs, and we then used systematic random sampling to target every 8^th^ (3,287/400) patient from the EHR list.

### Instruments

The LATCon II [11] is a 20-item scale measured using a 4-point Likert scale: strongly disagree (0), disagree (1), agree (2), and strongly agree (3). Five items (i.e. Items 11, 14, 15, 18, and 20) in the original English LATCon II were reversed scored, and overall the original English instrument had satisfactory psychometric properties: test-retest reliability (Pearson’s correlation coefficient = 0.64), internal consistency reliability (Cronbach’s α = 0.82), and construct validity [11]. Exploratory factor analysis (EFA) identified five components, which explained 54 % of the overall variance. The components were: 1) level of participation and involvement that the respondents feel patients’ should have within the consultation; 2) continued use of a paternalistic style of interaction; 3) necessity to find common ground and be in agreement over decisions; 4) perceived benefits of partnership in medicine taking; and 5) equality and shared control within the interaction. By translating the LATCon II into Chinese language will enable the attitudes towards concordance during health communication to be measured in Chinese speaking patients.

This study used the 25-item 5-point Likert Therapeutic Adherence Scale for Hypertensive Patients (TASHP) [20] to measure the level of adherence to treatment to test the criteria-related validity. This instrument was already available in Chinese language. The TASHP measures patients’ health behaviours required to manage hypertension, whereas the LATCon II measures their attitudes. In this study the criteria-related validity was based on the theoretical linkage between health communication and health behaviours [21, 22].

### Data collection

Data were collected between March and August 2013.

#### Stage 1: translation process

**Fig. 1:**
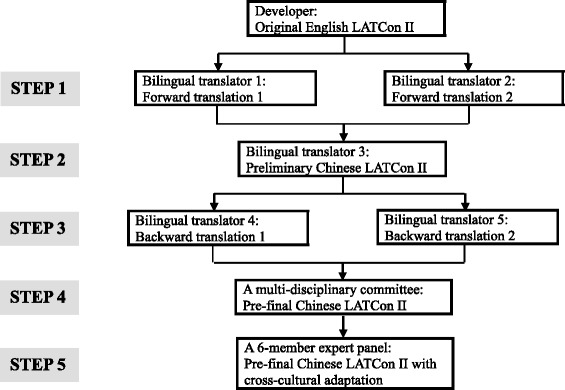
Translation process

The process for translating the LATCon II from English into Chinese followed Sousa’s guidelines of translation, adaptation and validation of instruments [19] and is summarised in Fig. 1. The translation technique contained five steps: 1) A forward translation was conducted by two independent translators with distinct backgrounds from source language (English) to target language (Chinese); 2) A third independent translator initially compared the two forward-translated versions of the LATCon II with the original version of the LATCon II regarding ambiguities and discrepancies of words, sentences and meanings. The second step generated a preliminary initial translated version of the LATCon II in Chinese; 3) The initial Chinese version of the LATCon II was translated back into English by two other independent translators with the same qualifications and characteristics, described above in step one. These two translators were blind to the original English version of the LATCon II. This process resulted in two back-translated versions of the LATCon II in English; 4) A multi-disciplinary committee was established by the above five translators to discuss the ambiguities and discrepancies between the two back-translations and between each one of the two back-translations and the original LATCon II in English. The developer of the original English LATCon II was invited to resolve any confusion on the meaning of the items. This step established content equivalence between English and Chinese versions of the LATCon II to generate the pre-final Chinese version of the LATCon II; and 5). An expert panel with six members were invited to conduct cross-cultural adaptation for the pre-final version.

The selection criteria of five translators were: 1) fluent in English and experienced in translation; 2) holding the title of Associate Professor or Co-Chief nurse or higher; and 3) with educational background of master’s degree or higher. The selection criteria of six expert panel members were: 1) experienced in health communication during nursing practice; 2) holding the title of Associate Professor or Co-Chief Nurse or higher; 3) with educational background of Master’s Degree or higher; and 4) specialised in psychometric evaluation.

#### Stage 2: pilot study

The pre-final version of the instrument was pilot tested among Chinese hypertensive patients to evaluate the instructions, response format and the items of the LATCon II for clarity. Using convenience sampling from the patients at the CHCs, fifteen participants were invited to rate the instructions and items of the LATCon II using a dichotomous scale (clear or unclear). Any participants who rated the instructions, response format or any item of the LATCon II as unclear were asked to provide suggestions on the modification. The Chinese version of the LATCon II (C-LATCon II) scale was then finalised. Then, the six-member expert panel was invited to evaluate each item of the instrument for content equivalence using the following scale: 1 = not relevant; 2 = unable to assess relevance; 3 = relevant but needs minor alteration; 4 = very relevant and succinct.

#### Stage 3: primary cross-sectional survey

Following training by the first author, five CHC health professionals recruited potential participants through initial telephone contact using a standard script to avoid perceived coercion. A random sample of interview was selected to monitor adherence to study protocol and to ensure fidelity. The patients who agreed to participate in this study were then invited to come to the CHCs for a routine health appointment. The rationale for this procedure was that patients were familiar with and trusted the CHC health professionals who were responsible for their healthcare. After telephone recruitment, further and detailed explanation was provided by the first author at the CHC. Participants then provided voluntary consent before completing the C-LATCon II and the TASHP. In addition, 30 participants were invited to complete the C-LATCon II 14 days (time 2) after their first participation (time 1). The scales were checked for any missing data after the participants’ completion when they submitted to investigators. Once missing data were found, the scales were given back to the participants to complete.

### Data analysis

In the pilot study, first, the interrater agreement (IR) among patients was calculated to evaluate the level of their understanding of the C-LATCon II. Then, IR among expert panel members was calculated to reflect their proportion of agreement on evaluating the C-LATCon II [23]. After that, content validity of the C-LATCon II was tested by measuring the content validity index (CVI), including item-level CVI (I-CVI) and scale-level CVI (S-CVI). S-CVI includes S-CVI/UA (universal agreement) and S-CVI/Ave (averaging calculation). Modified kappa statistic (k*) was used to examine the agreement on each item which was rated ≥ 3 by the expert panel members. The criteria for a satisfactory CVI used in this study were: IR ≥ 0.8; I-CVI ≥ 0.83; k* ≥ 0.74; S-CVI/UA ≥ 0.8; and S-CVI/Ave ≥ 0.9 [24, 25].

In the primary cross-sectional survey, participants who did not complete half of the items in the three scales were deleted from the raw data. Little’s Missing Completely at Random (MCAR) Test was used to evaluate whether the continuous data were missed randomly; revealed a *p* value > 0.05 indicating that data were missed in a random way. Expectation Maximization (EM) was used to impute missing continuous data. Mode substitution was used to impute missing categorical data.

Data were analysed using Statistical Package for Social Sciences (SPSS) 21.0 for Windows. Construct validity was tested by conducting EFA. Criteria-related validity was tested by measuring Pearson’s correlation between the C-LATCon II and the TASHP. Internal consistency was tested by calculating Cronbach’s α. Test-retest reliability was calculated by Pearson’ correlation coefficient and intraclass correlation coefficient (ICC) between time 1 and time 2, and by linear weighted Cohen’s kappa statistic for each item. Two-sided *p* < 0.05 was considered statistically significant.

### Ethical considerations

Permission was requested from participants and the manager in charge of the CHCs selected. Approvals were received from Nantong University Affiliated Hospital Ethics Committee [2012(030)] and Queensland University of Technology Ethics Committee (1300000022).

## Results

### Stage 1: translation process

To make the questions in the LATCon II contextually suitable for Chinese hypertensive patients, three changes were made after translation and before the pilot study was conducted. First, all of the words “patients” in the original 20-item LATCon II were replaced with “me”. Second, in the 5th step of the instrument translation process, the expert panel recommended deleting Item 19: “During the doctor-patient consultation my decision is the most important” and Item 20: “I should be able to take on as much responsibility as I wish for my own treatment” due to the highly likely confusion and misunderstanding among the target (Chinese) population. This change yielded a total number of 18 items in the pre-final C-LATCon II. The two deleted items were also translated in the pilot study to seek participants’ understanding of and suggestion regarding these items. Third, “medicine” in three items of the original LATCon II were changed into “treatment” for the following two reasons: 1) “the benefits of the medicine” in Item 8, “medicine taking” in Item 13, and “patients’ views about medicines” in Item 14 refer to only medication therapy in Chinese language, while hypertension management includes not only medication therapy but also lifestyle modifications; and 2) similarly, “treatment” indicates both medication therapy and lifestyle modifications prescribed by health professionals.

### Stage 2: pilot study

The IR for the 18-item pre-final C-LATCon II was 100 %, which indicated that all participants (*n* = 15) clearly understood all 18 items of the pre-final C-LATCon II. Participants also expressed their confusion about the two deleted items. There were three primary reasons for their confusion: 1) they felt unclear about what kind of “decision” Item 19 refers to; 2) they felt confused about Item 19 because they thought that they would have not consulted doctors if their decision were the most important; and 3) they simply did not understand what Item 20 meant. As a result, Item 19 and Item 20 were removed prior to testing the psychometric properties. The C-LATCon II was then finalised and ready for psychometric testing. An additional file shows the C-LATCon II in more detail [see Additional file 1].

Before measuring CVI, the IR among expert panel members of 0.89 indicated a good proportion of agreement on evaluating the C-LATCon II [23]. I-CVI was 0.83-1. k* was 0.81-1. S-CVI/UA was 0.89. S-CVI/Ave was 0.98. All content validity indices showed that the C-LATCon II had a satisfactory content validity.

### Stage 3: primary cross-sectional survey

Of all the 411 (3287/8) potential participants contacted by telephone, eight cases were excluded (not meeting inclusion criteria), 21 cases did not respond, and 382 cases agreed to participate at the CHCs. After telephone recruitment, 13 cases did not attend their appointments at the CHCs, and 369 participants completed the demographic characteristics, C-LATCon II and TASHP. The data for three participants were deleted because they had completed less than half of the items of the instruments, yielding a final total number of 366 participants. No missing data were found in the items of the C-LATCon II and the TASHP.

#### Descriptive analysis of demographic characteristics and the C-LATCon II

The participants recruited in this study were on average 67 years of age. The number of males (*n* = 186) was almost equal to that of females (*n* = 180). Most of the participants were married (90.2 %). The demographic characteristics are summarised in Table 1. The total possible score of the C-LATCon II was 54. In this sample, the mean and standard deviation (SD) were 38.83 ± 4.72.

**Table 1:** Demographic and socioeconomic characteristics (*n* = 366)

Characteristics	No. of Missing data (%)	Mean ± SD or Frequency (%)
Age	6 (1.6)	66.91 ± 9.33
Gender	0	
Male		186 (50.8)
Female		180 (49.2)
Marital status	1 (0.3)	
Married		330 (90.2)
Others		35 (9.5)
Education	1 (0.3)	
Primary school or lower		63 (17.2)
Junior high school		98 (26.8)
Senior high school/certificate/diploma		146 (39.9)
Undergraduate or higher		58 (15.8)
Employment	3 (0.8)	
Employed		49 (13.4)
Unemployed		6 (1.6)
Retired		297 (81.1)
Farming		11 (3.0)
Duration of hypertension (years)	3 (0.8)	
<1		18 (4.9)
1 – 9		132 (36.1)
10 – 19		135 (36.9)
≥20		78 (21.3)

#### Construct validity

Six assumptions for EFA were tested before conducting EFA [26]. First, multivariate outliers (*n* = 13) were detected and then deleted by calculating Mahalanobis distance and using the criterion of α = 0.001 with 18 *df* (the critical *χ*^2^ = 42.31). Second, the finalised sample size of 353 (366–13) in this study was sufficient to conduct EFA for the 18-item C-LATCon II. A sufficient sample size minimises the probability of errors and maximises the accuracy of population estimates. Third, multicollinearity and singularity were tested by calculating *tolerance* value and variance inflation factor (*VIF*). The *tolerance* values were between 0.31 − 0.76. The *VIF* values were between 1.32 − 3.22 (<5.0). So, the assumption of absence of multicollinearity and singularity held. Fourth, the Shapiro-Wilk normality test showed that none of the C-LATCon II items were normally distributed (*p* < 0.05). When examining the raw data, most participants scored 2 or 3 points in the items of this 4-point (from 0 to 3 points) Likert scale. The Shapiro-Wilk normality test is not appropriate to test the normality of Likert items when participants seldom score items at either end of the scale. However, the skewness and kurtosis values of all 18 items varied between −0.33 − 0.33 and −1.78 − 0.63 respectively which indicated that normality was not a severe issue. Even so, Principal Components Analysis (PCA) was chosen to extract components in this study because PCA is robust on non-normality data. Fifth, linearity among pairs of variables was assessed through measurement of correlation. It was impractical to examine all pairwise correlation to test linearity for all 18 items. So, this study chose to test the correlation between the item with the strongest positive skewness (0.33) and the item with the strongest negative skewness (−0.33) because the linearity between two opposite extreme items was expected to be among the worst. The Pearson’s correlation coefficient was 0.34 (*p* < 0.001). This result indicated that there was no evidence of true curvilinearity. Sixth, the Kaiser-Meyer-Olkin (KMO) Measure of Sampling Adequacy value of 0.84 (>0.6) and Bartlett’s Test of Sphericity (*χ*^2^ (153) = 2213.83, *p* < 0.001) indicated that the data were suitable for EFA.

**Fig. 2:**
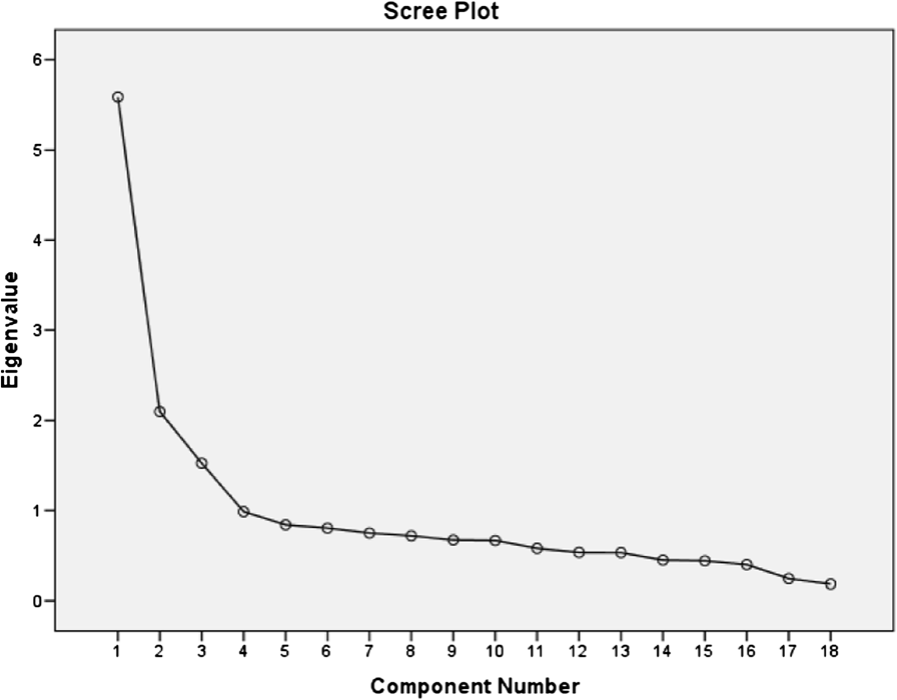
Scree test

The number of components was determined by combining three criteria: Cattell’s scree test, Kaiser’s eigenvalue-greater-than-one rule, and Horn’s parallel analysis [27]. The scree plot indicates that a sharp drop in the plot occurred between the third and fourth lines and therefore suggested a four-component extraction (Fig. 2). Four components had eigenvalues ≥ 1, which suggested that the number of components extracted was four (Table 2). According to the output of *Monte Carlo* PCA for parallel analysis, the Component 3 was considered significant because the eigenvalue (1.53) of the Component 3 was bigger than the 3^rd^ random eigenvalue (1.27), whilst the Component 4 was considered insignificant because the eigenvalue (1.00) of the Component 4 was smaller than the 4^th^ random eigenvalue (1.22) (Table 2). Although the scree plot, eigenvalue rule and parallel analysis provided inconsistent results of the number of components to be extracted, four components were identified after considering that the components extracted should reasonably interpret the items. These components accounted for more than 56 % of the total variance (Table 2).

**Table 2:** Means, SDs, factor loadings, and communalities of C-LATCon II (loadings ≥ 0.35 are shown, *n* = 353)

Factors	Item	Item content	Mean (SD)	C1	C2	C3	C4	*h* ^2^
Health professionals’ attitude	1	Prescribing should take account of my expectations of treatment	2.26 (0.56)	0.72				0.54
	5	Doctors should try to help me to make as informed a choice as possible about benefits and risks of alternative treatments	2.52 (0.50)	0.53				0.44
	7	Doctors should give me the opportunity to talk through my thoughts about my illness	2.35 (0.53)	0.81				0.58
	8	Doctors should make clear when the benefits of the treatment are uncertain	2.43 (0.50)	0.46				0.45
	9	Doctors should be more sensitive to how I react to the information they give	2.40 (0.50)	0.50				0.46
	10	It is always important for doctors to listen to my personal understanding of my condition	2.43 (0.53)	0.70				0.51
	13	Doctors should encourage me to express my concerns about treatment	2.34 (0.54)	0.74				0.56
	18	It is not always necessary for doctors to take account of my priorities	2.25 (0.53)	0.53				0.36
Partnership between two parties	12	The doctor and I should find common ground on what the problem is and jointly agree on what to do	1.20 (0.75)		0.91			0.86
	15	The doctor is the expert and my role is to do as the doctor says	1.11 (0.73)		0.75			0.68
	16	The consultation between the doctor and I should be viewed as a negotiation between equals	1.20 (0.70)		0.78			0.67
Therapeutic decision making	2	Doctors and I should agree a treatment plan that takes account of both views	2.40 (0.51)			−0.79		0.68
	6	During the consultation both the doctor and I should state views about possible treatments	2.32 (0.52)			−0.76		0.62
	17	A good treatment decision is made when both the doctor and I agree on the treatment to use	2.39 (0.54)			−0.96		0.85
Patients’ involvement	3	My involvement in the prescribing process always leads to better outcomes	2.32 (0.57)				0.36	0.41
	4	The best use of treatments is when it is what I want and am able to achieve	2.39 (0.55)				0.47	0.45
	11	It is sometimes appropriate for the doctor to make treatment decisions without my input	2.25 (0.56)				0.65	0.47
	14	Taking account of my views about treatment is not always necessary for appropriate prescribing	2.30 (0.60)				0.73	0.60
Eigenvalue				5.59	2.10	1.53	1.00	
Percentage of variance explained (%) (cumulative 56.66)				31.04	11.66	8.48	5.48	
Cronbach’s α (total 0.78)				0.84	0.66	0.78	0.78	

Items 11, 14, 15, and 18 are reversely scored. C 1–4 = Component 1–4. *h* ^2^ = communality

Factor rotation was conducted for further interpretation. Direct oblimin rotation was chosen because correlation was assumed between the extracted components in the C-LATCon II. Table 2 shows the four components extracted and the factor loading of each item. The four components were defined as: 1) health professionals’ attitudes; 2) partnership between two parties; 3) therapeutic decision making; and 4) patients’ involvement.

#### Criteria-related validity

Normality of the two scales was measured before calculating Pearson’s correlation coefficient between the C-LATCon II and the TASHP. According to the Shapiro-Wilk test, both scales were not normally distributed (*p* < 0.05). Because the Shapiro-Wilk test may be statistically significant from a normal distribution in large samples [28], graphic distribution was required for verification. According to the histograms, the two scales were roughly bell shaped. So, there was no severe problem of normal distribution in the two scales. Although the Pearson’s correlation coefficient was 0.11 and statistically significant (*p* < 0.05), the small value of the Pearson’s correlation coefficient showed that in this study sample the direct association between patients’ attitudes towards concordance during health communication and their health behaviour was weak.

#### Internal reliability

The value of Cronbach’s α of overall scale was 0.78, indicating that the C-LATCon II had good internal reliability (Table 2). The component “partnership between two parties” had the lowest Cronbach’s α at 0.66 which is close to 0.7 and was therefore acceptable. The Cronbach’s α of the other three components were over 0.7, showing satisfactory internal reliability [29].

#### Test-retest reliability

**Fig. 3:**
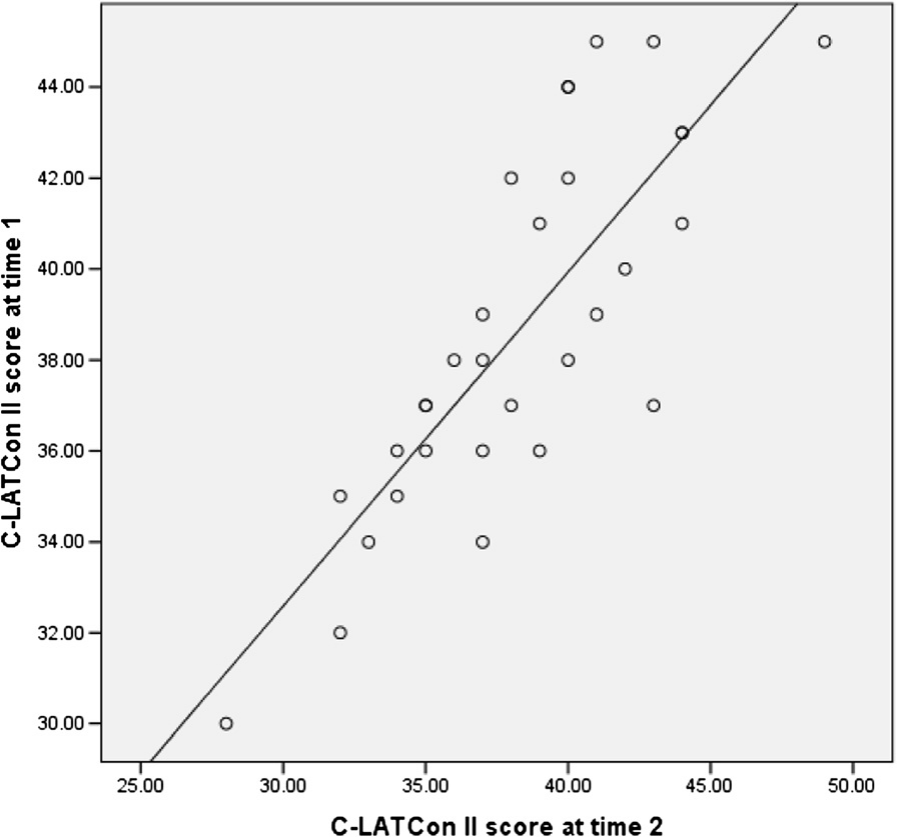
Scatter plot indicating total scale scores at time 1 and time 2 (*n* = 30)

Thirty participants completed the C-LATCon II at time 1 and time 2 (14 days after time 1). Shapiro-Wilk’s normality test of the overall C-LATCon II at time 1 (*p* > 0.05) and time 2 (*p* > 0.05) indicated a normal distribution at both times. Pearson’s correlation coefficient of 0.82 (*p* < 0.001) and ICC (one-way random, single measures) of 0.82 (*p* < 0.001) indicated a statistically significant and positive association of the total C-LATCon II scores between time 1 and time 2 (Fig. 3). The values of linear weighted kappa statistic for each item of the C-LATCon II varied between 0.40-0.65 (*p* < 0.05), which indicated that there was a mostly moderate agreement (0.41-0.60 = moderate; 0.61-0.80 = substantial) for each item at the different time points [30]. Because the C-LATCon II is a self-reported instrument with four response options for each item, the results of linear weighted kappa statistic showed some variation in responses for each item at different time points. However, the results of Pearson’s correlation coefficient and ICC suggested a satisfactory test-retest reliability of the C-LATCon II.

## Discussion

This study translated the C-LATCon II, and reported satisfactory psychometric properties of the scale by measuring content validity, construct validity, internal reliability, and test-retest reliability. This study revealed that the C-LATCon II appeared to be a reliable and validated instrument, which can be used to measure patients’ attitudes towards concordance during health communication in Chinese population. This study was unique in three characteristics. First, the translation process from the original English version of LATCon II to the target Chinese version of LATCon II rigorously followed Sousa’s guidelines to ensure that equivalence between the two versions was established. Second, a systematic random sampling was used to recruit participants. The purposes for this sampling strategy were to assist with both the representativeness of the sample and the generalisation of the results to a Chinese population. Third, this study for the first time tested content validity and criteria-related validity of the concordance instrument. It should be noted that the test of criteria-related validity showed that patients’ attitudes towards concordance during health communication might not be an independent factor influencing their health behaviours. Other factors (e.g. knowledge of hypertension management and self-efficacy of hypertension management) ought to be considered when testing the association between patients’ attitudes towards concordance during health communication and their health behaviours in future research.

A four-component structure was identified from the C-LATCon II. These components were consistent with Horne and Weinman’s three-phase (input factors, process of health communication, and outcome of health communication) framework of concordance during health communication [31]. The component *health professionals’ attitude* described patients’ expectation of health professionals’ attitudes towards health communication. The component *patients’ involvement* referred to patients’ attitudes towards their involvement in health communication. Both the first two components reflected the patients’ perception of the attitudes from two parties before commencement of health communication, and therefore belonged to input factors. The third component *partnership between two parties* described how patients perceived the relationship between two parties during the encounter, and therefore revealed how patients looked at the process of health communication. The fourth component *therapeutic decision making* described the patients’ attitudes towards the roles played by both parties when deciding treatment regimens, and demonstrated the outcome of health communication. So, these four components accurately measured patients’ attitudes towards health communication at the three different phases of Horne and Weinman’s framework.

The components extracted in this study were different from the five components in the original UK study [11], the three components in a Spanish study [12], and the four components in a US study [13]. There are three potential reasons for the difference of the components. First, the different parties recruited might account for differences in the components extracted. For example, in the UK study, student nurses, medical students, and pre-registration pharmacists at the same UK University were recruited. The Spanish study recruited psychiatrists and psychiatry registrars. The US study recruited students enrolled in nursing, respiratory care and pharmacy. So, these three studies recruited health professionals as the participants. In contrast to the previous three studies, this present study targeted patients instead of health professionals as the participants.

The second reason is due to the different ethnic populations recruited in these studies. The three previous studies recruited participants from UK, Spanish and US populations, while the current study recruited Chinese participants. No previous studies have been conducted to investigate patients’ attitudes to concordance by using the LATCon II, so it is not possible to compare the influence of culture on patients’ attitudes to concordance with other cultural populations. However, in the pilot study, Chinese participants’ failure to understand two of the translated items from the original English LATCon II reflects the different perception of concordance between cultural groups.

Third, the deletion of two items from the original LATCon II influenced the component extraction. From a statistical perspective, a change in the number of items in a scale will potentially change the number of components and the items within components extracted via EFA.

Patients with an expectation of a collaborative attitude from health professionals and active involvement in health communication tend to expect to negotiate their healthcare as an equal (i.e. their input is valued by health professionals). Surprisingly, this study found that Chinese hypertensive patients scored the component *partnership between two parties* much lower than the other three components (*health professionals’ attitude, patients’ involvement,* and *therapeutic decision making*). That is, patients in this study tended to recognise an unequal partnership with health professionals, but they also expected at the same time a collaborative attitude from health professionals and being involved in therapeutic decision making. There might be two reasons for the perception of accepting an unequal partnership with health professionals. First, health professionals’ traditional paternalistic attitudes to health communication makes Chinese patients believe that the partnership is unequal [32, 33]. Second, the long-term unequal partnership makes Chinese patients habitually believe that health professionals should dominate the way health communication occurs.

### Limitation and future implications

This study recruited participants from nine CHCs, although all CHCs came from one area in China due to budgetary constraints. The systematic random sampling conducted in this study might have potential unknown biases. A future multi-centre study should be conducted by recruiting participants from more extensive regions in China and using stratified random sampling. Further research might be conducted to investigate the attitudes towards concordance during health communication in patients with other chronic diseases. The C-LATCon II could be used to measure the effect of interventions designed to improve patients’ attitudes towards concordance during health communication in future research. Nevertheless the rigour of the translation process and testing procedures were a strength of this study.

## Conclusion

The C-LATCon II is a validated and reliable instrument which can be used to evaluate patients’ attitudes towards concordance during health communication in Chinese population. The four components (health professionals’ attitude, partnership between two parties, therapeutic decision making and patients’ involvement) can be used to describe patients’ attitudes towards concordance during health communication.

## Additional file

Additional file 1:
**The Chinese version of the Leeds Attitudes to Concordance II scale.** (PDF 75 kb)

## Abbreviations

C-LATCon II: The Chinese version of the Leeds Attitudes to Concordance II scale
CHC: Community Health Centre
EFA: Exploratory factor analysis
EHR: Electronic Health Record
EM: Expectation maximization
I-CVI: Item-level content validity index
ICC: Interclass correlation coefficient
IR: Interrater agreement
MCAR: Little’s missing completely at random
PCA: Principal components analysis
S-CVI/Ave: Scale-level content validity index/averaging calculation
S-CVI/UA: Scale-level content validity index/universal agreement
TASHP: Therapeutic adherence scale for hypertensive patients
VIF: Variance inflation factor
