# Supplementary material for: Translation and psychometric properties of the Chinese version of the Leeds Attitudes to Concordance II scale
Source: BMC Med Inform Decis Mak. 2015 Aug 1;15:60. doi: 10.1186/s12911-015-0184-0 (PMC4522111; doi:10.1186/s12911-015-0184-0)
Supplement: Additional file 1: — The Chinese version of the Leeds Attitudes to Concordance II scale. (PDF 75 kb) [file 12911_2015_184_MOESM1_ESM.pdf]

下列问题关于您是如何理解医护人员和您的沟通的？本问卷的答案没有对错之分，请在最能反映您的观点的选项下面划**“√”**

| **编号** | **问题** | **完全不同意** | **基本不同意** | **基本同意** | **完全同意** |
| --- | --- | --- | --- | --- | --- |
| 1 | 医护人员在制定治疗方案时，应该考虑到我对治疗的期望。 |  |  |  |  |
| 2 | 如果治疗方案包含了医护人员和我双方的观点，双方应该同意这个治疗方案。 |  |  |  |  |
| 3 | 有我参与制定的治疗方案，治疗效果会更好。 |  |  |  |  |
| 4 | 最好的治疗方案，既是我所想要的，又是我能够做到的。 |  |  |  |  |
| 5 | 关于治疗方案的好处和风险，医护人员应该尽可能地跟我说清楚，帮助我尽量在了解信息的基础上做出选择。 |  |  |  |  |
| 6 | 看病时，医护人员和我都应该对治疗方案提出各自的观点。 |  |  |  |  |
| 7 | 医护人员应当给我机会，让我谈谈对自身疾病的看法。 |  |  |  |  |
| 8 | 当治疗效果不明确时，医护人员就应该说明白。 |  |  |  |  |
| 9 | 我对医护人员所给信息的反应，医护人员应该更加关注。 |  |  |  |  |
| 10 | 医护人员倾听我对自身疾病的看法，这总是很重要的。 |  |  |  |  |
| 11 | 在没有我参与的情况下，医护人员决定了治疗方案，有时候这也是可以的。 |  |  |  |  |
| 12 | 医疗上有哪些问题，以及如何解决这些问题，医护人员应当和我达成一致。 |  |  |  |  |
| 13 | 医护人员应当鼓励我，让我谈谈对治疗的看法。 |  |  |  |  |
| 14 | 合理治疗方案的制定，不一定总要考虑我的观点。 |  |  |  |  |
| 15 | 医护人员是专家，我只要按照他们说的去做。 |  |  |  |  |
| 16 | 看病时，医护人员和我之间的协商应该是平等的。 |  |  |  |  |
| 17 | 医护人员和我都同意的治疗方案，是好的治疗方案。 |  |  |  |  |
| 18 | 我优先考虑的事情，医护人员不一定需要去考虑。 |  |  |  |  |
